# On the emergence of conductivity at SrTiO_3_-based oxide interfaces – an *in-situ* study

**DOI:** 10.1038/s41598-019-54463-w

**Published:** 2019-11-29

**Authors:** Merlin von Soosten, Dennis. V. Christensen, Chang-Beom Eom, Thomas. S. Jespersen, Yunzhong Chen, Nini Pryds

**Affiliations:** 1 Department of Energy Conversion and Storage, Technical University of Denmark, DTU Risø Campus, 4000 Roskilde, Denmark; 2 Center for Quantum Devices, Niels Bohr Institute, University of Copenhagen, 2100 Copenhagen, Denmark; 3 Department of Materials Science and Engineering, University of Wisconsin-Madison, Madison, Wisconsin 53706 United States

**Keywords:** Nanoscale materials, Electronic properties and materials

## Abstract

Heterostructures and crystal interfaces play a major role in state-of-the-art semiconductor devices and play a central role in the field of oxide electronics. In oxides the link between the microscopic properties of the interfaces and bulk properties of the resulting heterostructures challenge our fundamental understanding. Insights on the early growth stage of interfaces and its influence on resulting physical properties are scarce - typically the information is inferred from post growth characterization. Here, we report on real time measurements of the transport properties of SrTiO_3_-based heterostructures at room temperature, while the heterostructure is forming. Surprisingly, we detect a conducting interface already at the initial growth stage, much earlier than the well-established critical thickness limit for observing conductivity *ex-situ* after sample growth. We investigate how the conductivity depends on various physical processes occurring during pulsed laser depositions, including light illumination, particle bombardment by the plasma plume, interactions with the atmosphere and oxygen migration from SrTiO_3_ to the thin films of varying compositions. We conclude that the conductivity in these room-temperature grown interfaces stem from oxygen vacancies with a concentration determined primarily by a balance between vacancy formation through particle bombardment and interfacial redox reaction and vacancy annihilation through oxidation. Using this approach, we propose a new design tool to control the electrical properties of interfaces in real time during their formation.

## Introduction

Since the discovery of a two-dimensional electron gas (2DEG) at the interface between two band insulating oxides, SrTiO_3_ (STO) and LaAlO_3_ (LAO)^1^, a wealth of intriguing properties have emerged in this seemingly simple system. In the wake of LAO/STO, numerous other STO-based heterostructures has been formed by deposition of various oxide films on STO^2–4^. A common feature is that the properties of the interfaces can be tuned dramatically in numerous ways such as by controlling oxygen vacancies during growth and post annealing^5,6^, ion bombardment^7^, electrostatic gate potentials^8–10^, strain^11^, surface adsorbates^12,13^ and light exposure^14^. Pulsed laser deposition (PLD) remains the most popular deposition technique for growing STO-based heterostructures, but during this complex deposition process, STO is exposed to all the aforementioned stimuli. The laser shoots on the target and produces a plasma with an intense self-emission of visible and ultraviolet light^15^. The particles in the plasma plume travel towards the STO substrate where they impact with high kinetic energies on the order of tens of eV^15,16^ and produce a large and dynamically varying electrostatic surface potential^17^. As the particles arrive at the STO surface, they condense into a film that exerts stress onto STO and allows for mass transfer of e.g. oxygen ions across the interface^2^. Lastly, the entire process occurs in deposition conditions which opens up for exchange of oxygen with the atmosphere^18^ as well as adsorption of species such as water on the sample^19^. Complex processes are therefore expected to happen during the early stages of the growth, which may be of significant importance for the properties of the final film. If these processes can be understood and controlled, they will provide a new handle for tuning the interface properties in real time. This highlights the importance of studying the growth process in detail, but to date, only a few studies aim to partly analyze these processes^20,21^. The major difficulty lies in the limited number of techniques capable of monitoring and controlling the interface in real time at the early stage of the nucleation and growth. Reflection high-energy electron diffraction (RHEED) is commonly employed to monitor the film growth, to control the film thickness, and probe the crystallinity during growth in real-time^22^. It is, however, limited to structural information when growing crystalline materials. A supplementary way of monitoring the interfaces during growth is by measuring the resistivity of the interface during the growth process *in-situ*^20,21^. We expect that the combination of *in-situ* methods such as RHEED and conductivity measurements during growth will give access to a territory where the initial growth conditions can be studied and controlled in detail, leading to new and improved properties of the interface.

Here, we measure the sheet resistance of STO-based heterointerfaces and patterned devices in real time continuously from the early stage of the deposition until the final heterostructure is produced. The measured interface conductivity demonstrates the possibility to modulate the charge carriers at the interfaces in real time by engineering the top film and the oxygen content with an instant feedback on the properties.

**Figure 1:**
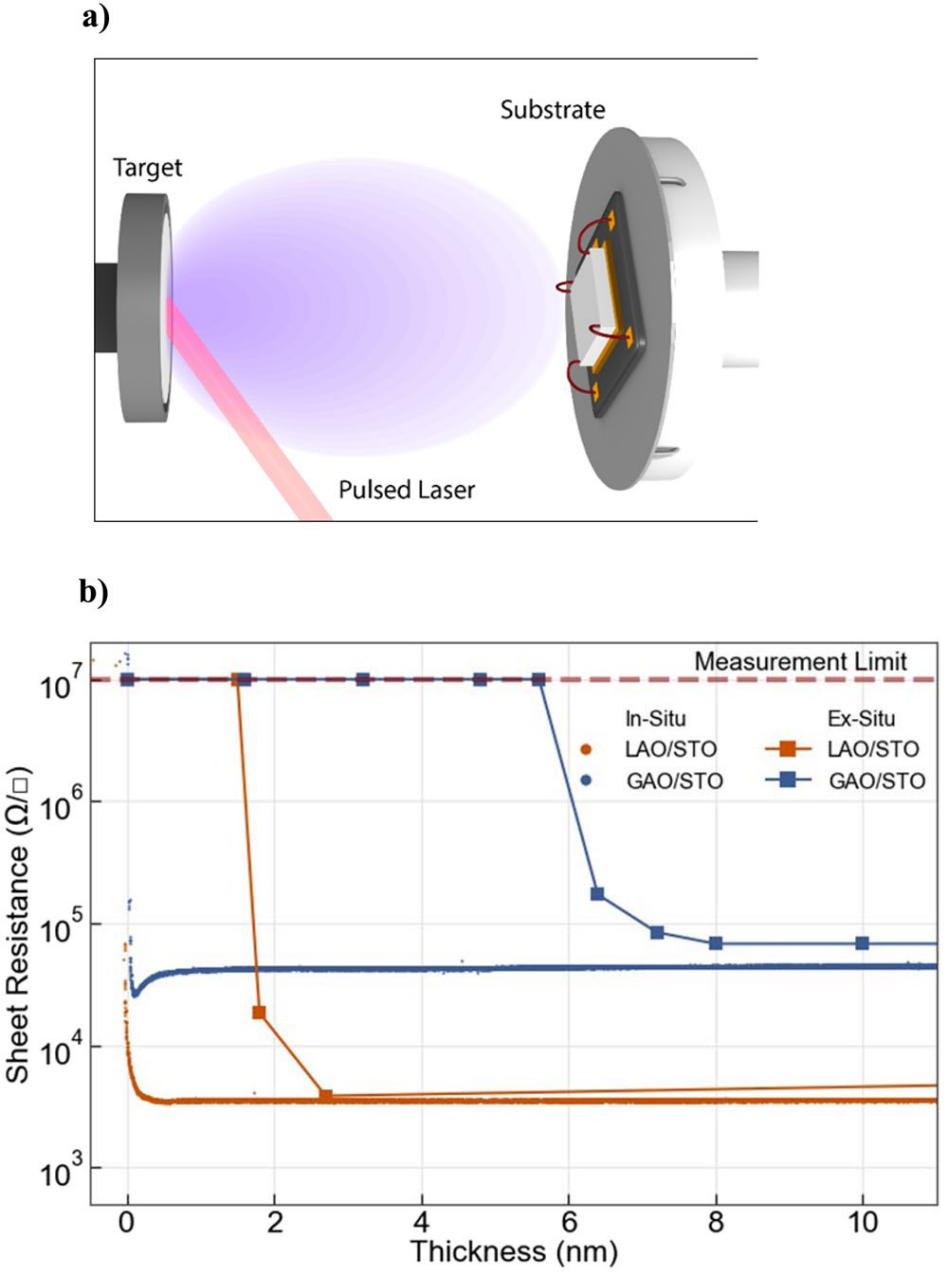
*In-situ* measurements of the interfacial sheet resistance during the deposition. (**a**) Schematic illustration of the *in-situ* transport measurement system in the PLD chamber. (**b**) Interface sheet resistance as a function of thickness for LAO/STO and GAO/STO measured *in-situ* and *ex-situ*. The *ex-situ* measurements were taken from^23^ with comparable deposition conditions, we note that the critical thickness, especially for GAO, does change with the deposition conditions^23^.

*In-situ* transport measurements were carried out inside the PLD chamber during room temperature film growth on TiO_2_ terminated STO (001) single crystals (See Fig. 1a). The evolution of the sheet resistance R_s_ (Ω/■) as a function of film thickness for LAO and γ-Al_2_O_3_ (GAO) deposited on STO is shown in Fig. 1b. The samples are initially insulating with R_s_  > 10^7^ Ω/■ (measurement limit), but after only a few laser pulses (<3 pulses) the sheet resistance drops to ~5 × 10^3^ Ω/■ and ~5 × 10^4^ Ω/■ for the LAO and the GAO top layers, respectively. As the deposition is continued (film thickness of 2 nm), the sheet resistance reaches a steady state value of 2 × 10^3^ Ω/■and 10^5^ Ω/■ for LAO and GAO top layers, close to the *ex-situ* measurements in similar samples^2,23^ (see also Fig. 1b for a comparison). Interestingly, at the initial growth stage (<2 nm thickness), the measured values of the interface sheet resistance are much lower than the *ex-situ* measurements.

**Figure 2:**
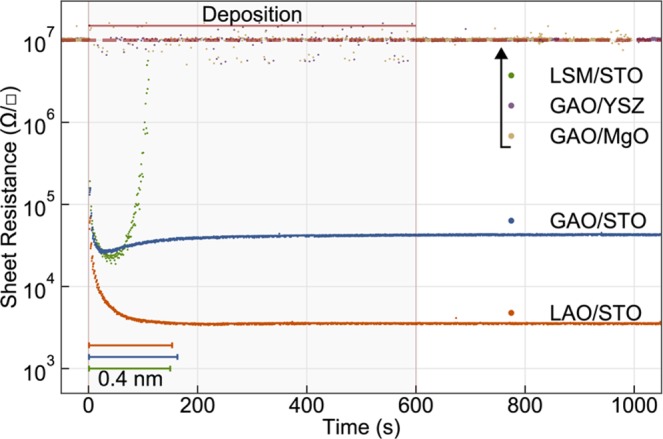
*In-situ* sheet resistance measurements. The measurements shown here are for the GAO/STO, LAO/STO, LSM/STO, GAO/MgO, GAO/YSZ heterostructures. Measurements on MgO and YSZ substrates remain at the measurement limit. The scale bar in the figure show the conversion of time to thickness for LAO, GAO and LSM.

A different behavior was found when LaSr_1/8_Mn_7/8_O_3_ (LSM) was deposited on STO as shown in Fig. 2. LSM/STO interfaces are well-known to be insulating as confirmed also in our *ex-situ* measurements after retrieving the samples from the PLD chamber. Surprisingly, however, the *in-situ* measurements reveal that the LSM films initially create conducting interfaces (<3 pulses) which turn insulating again as the growth progresses. Deposition of GAO was also performed on insulating MgO and yttria-stabilized zirconia (YSZ), and no conductivity was measured using these substrates (Fig. 2). This confirms that the charged species in the plasma are not contributing to the measured conductivity.

**Figure 3:**
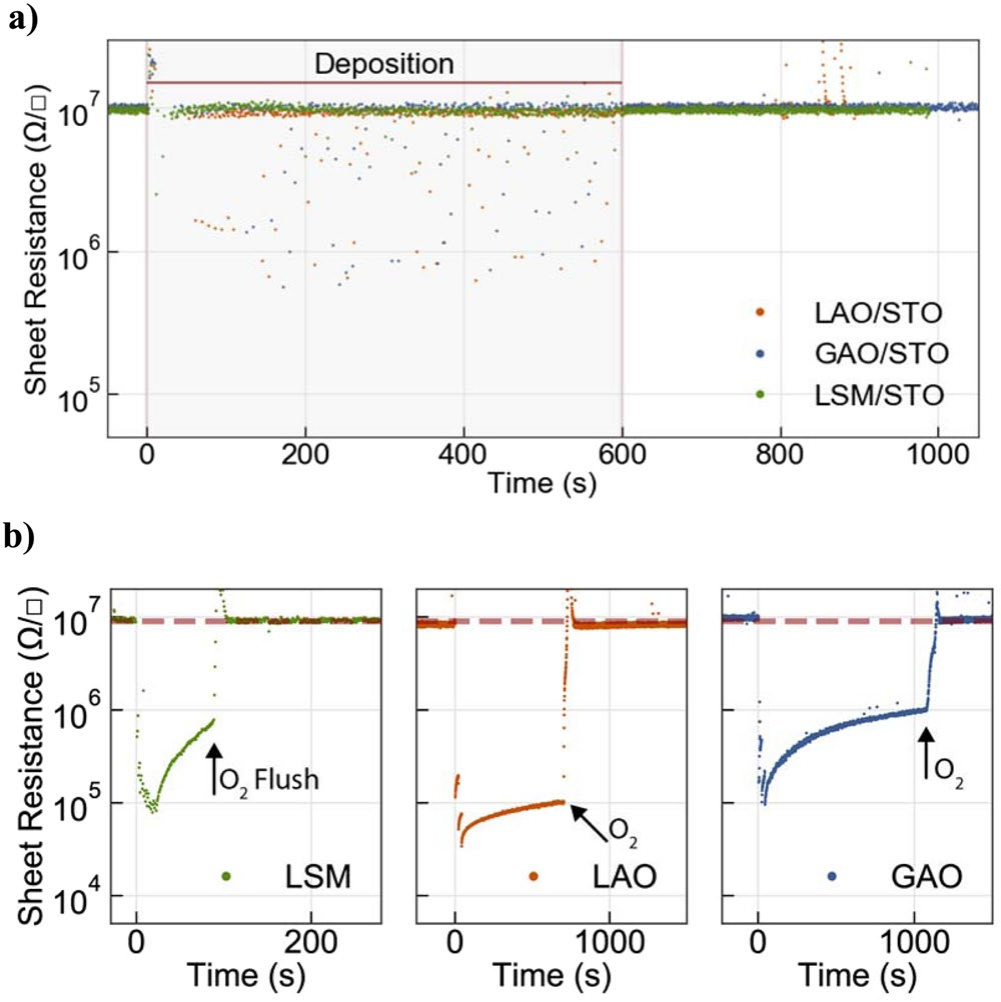
(**a**) No measurable conductivity change is found for Sapphire covered substrates, the conductivity remains at the measurement limit throughout the deposition time. (**b)** Annealing experiments illustrating the effect of oxygen on the sheet resistance. After 3 initial laser pulses samples turn conducting and decay in 2 × 10^−6^ mbar of Oxygen. The arrow in the figures show the onset of oxygen flushing to 5 × 10^−2^ mbar into the chamber, and the samples turn insulating rapidly.

STO and STO-based heterostructures have been reported to turn conductive when exposed to UV radiation^20,21,24–26^. To distinguish the light induced conductivity from other possible effects that can cause conductivity, we place a double-sided polished sapphire window in close proximity in front of the sample. The sapphire blocks only material from reaching the STO surface but not the UV or visible light. When the sapphire window is inserted, the deposition of LAO, GAO, and LSM show no detectable effects on treated STO substrates, and the sheet resistance remains above the measurement limit (see Fig. 3a). Also conducting samples with previously grown GAO or LAO films show no substantial effect during deposition with a sapphire window. Only samples with a high sheet resistance of 10^6^ Ω/■, show a small response to the irradiation (see Fig. S1). These samples were grown at room temperature, and subsequently annealed in air at 150 °C^5^ in order to increase the sheet resistance to 10^6^ Ω/■.

Several conducting STO-based interfaces have been grown at room temperature where the origin of the conductivity is attributed to oxygen vacancies^2,27,28^. In order to understand the effect of oxygen on the interfacial conductivity we carried out *in-situ* studies of the effect of background gas by flushing the PLD chamber with oxygen after the deposition, see Fig. 3b. Initially the laser was pulsed 3 times, and the resistance drops by several orders of magnitude, from 10^7^ to 10^4^ Ω/■. After the short deposition time, the laser was turned off and the conductivity decays in an oxygen background pressure of 2 × 10^−6^ mbar. Using an exponential fit to the conductance (see Fig.S2) we obtain decay rates of 0.005 s^−1^ (LAO), 0.006 s^−1^ (GAO), and 0.081 s^−1^ (LSM). Figure 3b also shows a sharp increase in sheet resistance to the detection limit, when flushing the chamber with oxygen to a pressure of 5 × 10^−2^ mbar. A similar resistance increase was not found when flushing the chamber in nitrogen. The modulation of the conductivity by alternating between depositing a few pulses and flushing the chamber with oxygen is repeatable (see Fig. S2). The degradation, however, diminishes with an increasing top film thickness, eventually forming heterostructures stable enough to be exposed to the ambient atmosphere.

Our annealing experiments reveal that the conductivity originates from oxygen vacancies, and hence the previously proposed mechanisms related to the polar discontinuity and La/Sr cation interdiffusion do not contribute to the emergence of conductivity during our room temperature depositions. This is consistent with the amorphous state of LAO and LSM (both lacking the crystalline order needed for the polar discontinuity to occur), the different transport behavior during LAO and LSM deposition (both containing La^3+^) and previous reports^2,5,28,29^. We therefore now consider the oxygen-vacancy related mechanisms that can influence the interface conductivity and explain our experimental findings. These mechanisms are bombardment^15^, light irradiation^14,20,21,24^, redox reaction^2,30–32^, and oxidation^5,27,29^ with three of them capable of forming conductivity.

### Bombardment

During the deposition, substrates are bombarded with high energy species from the target with kinetic energies on the order of tens of eV^15^, allowing loosely bound elements (i.e. oxygen) to escape from the topmost part of the substrate. This may form a conducting layer due to the formation of oxygen vacancies. During film growth, the substrate is increasingly protected by the deposited material, preventing the oxygen from leaving the interface, thus the bombardment effect is only relevant during the initial growth.

### Light Irradiation

STO absorbs light by exciting electrons across the bandgap at 3.2 eV or from in-gap states originating from, e.g., oxygen vacancies at lower energies. During deposition, free charges may therefore be generated and contribute to the conductivity of the sample.

### Redox reaction

During growth in a low oxygen pressure environment, an oxygen deficient film is generally formed, and for films with a high oxygen affinity a redox reaction may take place in which oxygen is transferred from the interface region of STO to the oxygen deficient top film. This results in the formation of oxygen vacancies as well as conductivity in STO. Similar to the bombardment, the redox reaction will only take place as long as there is enough energy and a pathway for oxygen to leave the substrate. This is dependent on the oxygen affinity of the top-film and the kinetics of the oxygen transfer, and the oxygen ions may in principle move either away from or into the top film depending on the energetic favorability.

In our current work we exclude any major contributions of light induced conductivity during the depositions as confirmed from our experiments using the sapphire window. This in contrast to previous reports^20^. Regardless of the film deposited on top of STO, all interfaces studied in the current work show a dramatic drop in resistance during the first laser pulses (See Fig. 2). Even LSM deposited on STO, which is reported to highly suppress the redox reaction and create non-conducting interfaces^2^, becomes conducting initially but decays very fast to its original high resistive state. Therefore, the measured conductivity after just 3 laser pulses cannot be attributed to the redox reaction only, but this initial change in conductivity is initiated by the bombardment with the plasma species. However, the bombardment cannot alone explain the transition to an insulating and higher resistive state when depositing LSM and GAO, respectively, and two additional, competing mechanisms are highly influential for determining the final conductivity of the interface.

The observed decay in the conductivity with time (see Fig. 3b), in particularly in the high oxygen pressure, reveal that oxygen available in the environment annihilates oxygen vacancies. This mechanism is supported by varying the film thickness: With thicker films the interface is protected better, resulting in lower decay rates for GAO and LAO consistent with a lower oxygen diffusion rates from the chamber to the interface across the top film. This also explains the difference between the *ex-situ* and *in-situ* measurements, as the initial conductivity is quenched by the atmospheric oxygen when taken out the chamber, if the top film has an insufficient thickness (smaller than the *ex-situ* critical thickness) to protect the oxygen vacancies in STO.

The transition from a metallic to a highly insulating state during the LSM deposition as well as the much higher decay rate after small amount of LSM deposition, however, suggest that the redox reaction plays an important role. The top film after 3 laser shots is estimated to cover only around 15% (based on the growth rate), and hence the direct oxidation from the 85% exposed surface should be comparable for the LAO, GAO and LSM deposition. However, taking into account the redox activity of the top film may explain this difference, as LSM can itself accommodate oxygen vacancies and electrons by changing the valence of Mn. This is consistent with several previous studies where both metal and oxide thin films containing only La and Al were found to have a much stronger reducing effect on STO compared to oxide or metal films containing Mn^2,30–32^.

Note that the role of oxygen vacancies in STO rather than oxygen vacancies in the top film is consistent with previous reports. First, *ex-situ* annealing^5,29^ yielded the conclusion of oxygen vacancies being present in STO as, e.g., the activation barrier of oxygen movement in STO was observed when quenching the conductivity by oxygen annealing in the case where the oxygen blocking layer GAO was deposited on STO^5^. Second, another study reported that an increase in the argon deposition pressure led to a metal-to-insulator transition in room temperature deposited LAO/STO despite a largely pressure-independent oxygen content was found in the plume (and hence presumably in the LAO film)^15^. This transition was attributed to a lowering of the kinetic energy of the plume species, leading to an insufficient activation of oxygen movement from STO to LAO.

In all depositions, we initially see the same effect: A sharp drop in resistance after only a few pulses due to oxygen vacancies created by bombardment. As the deposition progresses different top films show different behavior, and we therefore explain the differences by the following:

#### LAO/STO

The LAO film grown at room temperature has a high oxygen affinity, and oxygen is diffusing from STO to the amorphous film. The bombardment and redox reaction are dominating throughout the whole deposition process and the sheet resistance remains at a low level.

#### GAO/STO

The GAO film grown at room temperature is crystalline and the barrier for oxygen ion diffusion is higher than for LAO^5^. The deposition of GAO does not stabilize as many oxygen vacancies in STO as LAO, and with increased material deposition, oxygen diffusion is inhibited. Due to the competing mechanisms, the resistance increases after the initial low resistive state and stabilizes at a higher value.

#### LSM/STO

The effect of depositing LSM is markedly different from the other two and right after the initial stage where bombardment creates oxygen vacancies, the STO surface oxidizes due to the low oxygen affinity of LSM as well as interactions with the background gas. The sheet resistance recovers therefore quickly to its initial high resistive state.

**Figure 4:**
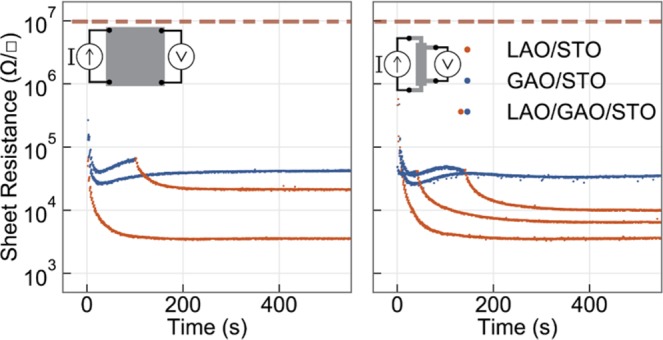
Tuning the sheet resistance of the interface via multilayers during the initial growth on (**a**) 5 × 5 mm samples and (**b**) patterned Hall-Bar (0.1 × 0.6 mm) samples. The sheet resistance in both geometries is comparable, as is the general behavior of multilayer depositions of LAO/GAO/STO. Red and blue data points mark measrements done during the deposition of LAO and GAO, respectively.

Based on the above results we were able to engineer the electronic properties of the interfaces at the atomic level by combining different sequences of materials deposited on the substrate. In Fig. 4 we show an example of the interface tunability by the combination of a sequence of deposited films such as LAO/GAO/STO. Here we combine the high quality epitaxial interface of GAO/STO with a top layer of LAO which results in a higher conductivity (compared to GAO/STO alone) due to the high carrier density caused by LAO deposition. By varying the amount of GAO deposited on the initial layer, we control the oxygen transfer to the LAO layer which gives a fine control on the final carrier density. This provides a simple and powerful tool to not only select desired properties arising from different materials, but also to fine-tune these properties due to the immediate feedback at the initial growth stage.

So far all of these experiments were done using a van der Pauw configuration (see method section). However, in order to confirm the robustness of the *in-situ* measurements, the experiments were repeated on UV-lithography patterned Hall-bar devices^33^, and no major differences were observed (see Fig. 4b).

In summary, we have shown that with a simple setup it is possible to monitor and study the sheet resistance of the interface in real-time during the early growth stage which is often not accessible during the growth process. Surprisingly, our results indicate that the conductive interface is created after the first few laser shots regardless the type of top film, in contrast to what can be deduced from *ex-situ* measurements after growth. The resulting interface conductivity is formed by a balance of bombardment, oxidation and redox reaction processes. This approach provides a new and yet undiscovered tool to tune the sample properties with real time feedback on the room temperature transport properties during the deposition by engineering the sequence of the materials deposited or by varying the deposition conditions. The control of the oxygen vacancy level can also be used to alter the low temperature transport properties, as a variation of the scattering landscape and amount of donors severely affects the carrier mobility and density at low temperatures^6,34^. This provides new opportunities to design interfaces in STO based heterostructures with controlled properties.

## Methods

### Methods

The interfaces were fabricated using 5 × 5 × 0.5 mm^3^ TiO_2_ -terminated SrTiO_3_ (001) single crystal substrates. Amorphous LaAlO_3_, amorphous LaSr_1/8_Mn_7/8_O_3_ and crystalline γ-Al_2_O_3_ layers were grown by PLD at an oxygen pressure of 2 × 10^−6^ mbar at room temperature, consistent with previous studies^2,9,35^. The thin films were grown by PLD using a KrF laser (λ = 248 nm) with a repetition rate of 0.5 Hz, and a laser fluence of 2.5 mJ cm^−2^. The target–substrate distance was kept constant at 40 mm. The film thickness and crystallinity were determined by RHEED oscillations (γ-Al_2_O_3_, see Fig. S3) and AFM measurements (LaAlO_3_, LaSr_1/8_Mn_7/8_O_3_ and γ-Al_2_O_3_). The electrical resistance of the interfaces was measured during the deposition process by means of a sample carrier constructed and placed inside the PLD chamber. The sample was electrically contacted by ultrasonic wire bonding with aluminum wire. Measurements were performed using a 4-probe method in the Van der Pauw geometry, except for Fig. 4b where the consistency between Hall-bar and Van der Pauw samples were studied. After verifying ohmic conductivity, the sheet resistance was extracted by linear fits to voltage biased I-V measurements with a maximum current of 50 nA and a repetition rate of 5 I/V traces per second. The linearity was occasionally perturbed by the deposition, and these outlying points were interpreted to result from interactions with the charge plasma and were not used. Permutations in the Van der Pauw geometry were verified throughout the experiments to show similar behavior, but the presented data was obtained in a single permutation to avoid dead time from permutation switching.

Hall-bar samples were prepared by UV-lithography, and PLD deposition was performed on the exposed and developed patterns^33^. A double-side polished sapphire plate was also placed in front of the samples, so that the sample could be shielded from the ablated particles. The sapphire glass were measured to be transparent to visible and UV light above 150 nm (the measurement limit of our transmittance measurement equipment). The conductivity was found to be invariant to whether the pressure gauge was turned on or off^19^ and whether the electron beam from the RHEED (20 kV, 1.55 A) irradiated the sample surface.

## References

[1] Ohtomo A, Hwang HY (2004). A High-Mobility Electron Gas at the LaAlO_3_/SrTiO_3_ Heterointerface. Nature.

[2] Chen Y (2011). Metallic and Insulating Interfaces of Amorphous SrTiO_3_-Based Oxide Heterostructures. Nano Letters.

[3] Chen YZ (2013). A High-Mobility Two-Dimensional Electron Gas at the Spinel/Perovskite Interface of γ-Al_2_O_3_/SrTiO_3_. *Nature*. Communications.

[4] Zhang, M. *et al*. Origin of Interfacial Conductivity at Complex Oxide Heterointerfaces: Possibility of Electron Transfer from Water Chemistry at Surface Oxygen Vacancies. *Physical Review**Materials*, 2 (6), 10.1103/PhysRevMaterials.2.065002 (2018).

[5] Christensen DV (2017). Controlling the Carrier Density of SrTiO_3_-Based Heterostructures with Annealing. Advanced Electronic Materials.

[6] Christensen DV (2018). Electron Mobility in γ-Al_2_O_3_/SrTiO_3_. Physical Review Applied.

[7] Aurino PP (2015). Reversible Metal-Insulator Transition of Ar-Irradiated LaAl O 3 / SrTi O 3 Interfaces. Physical Review B.

[8] Thiel S, Hammerl G, Schmehl A, Schneider CW, Mannhart J (2006). Tunable quasi-two-dimensional electron gases in oxide heterostructures. Science.

[9] Christensen DV (2016). Electric Field Control of the γ-Al_2_O_3_/SrTiO_3_ Interface Conductivity at Room Temperature. Applied Physics Letters.

[10] Christensen DV (2013). Controlling Interfacial States in Amorphous/Crystalline LaAlO_3_/SrTiO_3_ Heterostructures by Electric Fields. Applied Physics Letters.

[11] Jalan B, Allen SJ, Beltz GE, Moetakef P, Stemmer S (2011). Enhancing the Electron Mobility of SrTiO_3_ with Strain. Applied Physics Letters.

[12] Bi F (2010). “Water-Cycle” Mechanism for Writing and Erasing Nanostructures at the LaAlO_3_/SrTiO_3_ Interface. Applied Physics Letters.

[13] Xie, Y., Hikita, Y., Bell, C. & Hwang, H. Y. Control of Electronic Conduction at an Oxide Heterointerface Using Surface Polar Adsorbates. *Nature Communications*, 2 (1), 10.1038/ncomms1501 (2011). 10.1038/ncomms1501 21988910

[14] Lei Y (2014). Visible-Light-Enhanced Gating Effect at the LaAlO_3_/SrTiO_3_ Interface. Nature Communications.

[15] Sambri A (2012). Plasma Plume Effects on the Conductivity of Amorphous-LaAlO_3_/SrTiO_3_ Interfaces Grown by Pulsed Laser Deposition in O_2_ and Ar. Applied Physics Letters.

[16] Sambri A, Amoruso S, Wang X, Granozio FM, Bruzzese R (2008). Plume Propagation Dynamics of Complex Oxides in Oxygen. Journal of Applied Physics.

[17] Trier F (2013). Controlling the Conductivity of Amorphous LaAlO_3_/SrTiO_3_ Interfaces by *in-Situ* Application of an Electric Field during Fabrication. Applied Physics Letters.

[18] Xu C (2016). Disentanglement of Growth Dynamic and Thermodynamic Effects in LaAlO_3_/SrTiO_3_ Heterostructures. Scientific Reports.

[19] Scheiderer, P. *et al*. Surface-Interface Coupling in an Oxide Heterostructure: Impact of Adsorbates on LaAlO_3_/SrTiO_3_. *Physical Review B*, 92 (19), 10.1103/PhysRevB.92.195422 (2015).

[20] Shibuya K, Ohnishi T, Uozumi T, Koinuma H, Lippmaa M (2006). An *in Situ* Transport Measurement of Interfaces between SrTiO_3_(100) Surface and an Amorphous Wide-Gap Insulator. Applied Surface Science.

[21] Hensling, F. V. E. *et al*. UV Radiation Enhanced Oxygen Vacancy Formation Caused by the PLD Plasma Plume. *Scientific Reports*, 8 (1). 10.1038/s41598-018-27207-5 (2018). 10.1038/s41598-018-27207-5 PMC5996021 29892095

[22] Rijnders GJ, Koster G, Blank DH, Rogalla H (1997). *In Situ* Monitoring during Pulsed Laser Deposition of Complex Oxides Using Reflection High Energy Electron Diffraction under High Oxygen Pressure. Applied physics letters.

[23] Chen Y. Z., Bovet N., Kasama T., Gao W. W., Yazdi S., Ma C., Pryds N., Linderoth S. (2013). Room Temperature Formation of High-Mobility Two-Dimensional Electron Gases at Crystalline Complex Oxide Interfaces. Advanced Materials.

[24] Schütz P (2017). Microscopic Origin of the Mobility Enhancement at a Spinel/Perovskite Oxide Heterointerface Revealed by Photoemission Spectroscopy. Physical Review B.

[25] Walker SM (2015). Carrier-Density Control of the SrTiO_3_ (001) Surface 2D Electron Gas Studied by ARPES. Advanced Materials.

[26] Santander-Syro AF (2011). Two-Dimensional Electron Gas with Universal Subbands at the Surface of SrTiO_3_. Nature.

[27] Liu ZQ (2013). Origin of the Two-Dimensional Electron Gas at LaAlO_3_/SrTiO_3_ Interfaces: The Role of Oxygen Vacancies and Electronic Reconstruction. Physical Review X.

[28] Gunkel F (2017). Thermodynamic Ground States of Complex Oxide Heterointerfaces. ACS Applied Materials & Interfaces.

[29] Trier F (2013). Degradation of the Interfacial Conductivity in LaAlO_3_/SrTiO_3_ Heterostructures during Storage at Controlled Environments. Solid State Ionics.

[30] Fu Q, Wagner T (2007). Interaction of Nanostructured Metal Overlayers with Oxide Surfaces. Surface Science Reports.

[31] Fu Q, Wagner T (2005). Metal/Oxide Interfacial Reactions: Oxidation of Metals on SrTiO_3_ (100) and TiO_2_ (110). The Journal of Physical Chemistry B.

[32] Chen YZ, Pryds N, Sun JR, Shen BG, Linderoth S (2013). High-Mobility Two-Dimensional Electron Gases at Oxide Interfaces: Origins and Opportunities. Chinese Physics B.

[33] Bjørlig AV (2018). Nanoscale Patterning of Electronic Devices at the Amorphous LaAlO _3_ /SrTiO _3_ Oxide Interface Using an Electron Sensitive Polymer Mask. Applied Physics Letters.

[34] Trier F, Christensen DV, Pryds N (2018). Electron Mobility in Oxide Heterostructures. Journal of Physics D: Applied Physics.

[35] Christensen DV (2019). Stimulating Oxide Heterostructures - a Review on Controlling SrTiO_3_-Based Heterointerfaces with External Stimuli. Advanced Materials Interfaces.

